# The influence of water safety knowledge on adolescents’ drowning risk behaviors: a framework of risk-protect integrated and KAP theory

**DOI:** 10.3389/fpubh.2024.1354231

**Published:** 2024-05-10

**Authors:** Shi Luo, Shulai Luo, Zhongyu Ren, Hui Zhang, Xinyu Li, Lian Liu

**Affiliations:** ^1^School of Physical Education, Southwest University, Chongqing, China; ^2^School of Physical Education, Hubei Minzu University, Enshi, Hubei, China; ^3^School of Physical Education, Huzhou University, Huzhou, China

**Keywords:** adolescents, water safety knowledge, drowning risk perceptions, drowning risk attitudes, drowning risk behaviors

## Abstract

**Introduction:**

Although previous research has examined the risk factors for drowning behavior among adolescents, it is unclear whether this association is influenced by water safety knowledge. This study aimed to examine whether water safety knowledge is associated with adolescents’ drowning risk behaviors and whether drowning risk perceptions and attitudes could have a chain mediating role in the association between water safety knowledge and adolescents’ drowning risk behaviors.

**Methods:**

This study included 7,485 adolescents from five Chinese provinces and cities. We used the Drowning Risk Behaviors Scales (DRBS) to evaluate the risk of drowning behaviors. The Water Safety Knowledge Scale (WSKS) was used to evaluate the competence level of water safety knowledge. The Drowning Risk Perceptions Scale (DRPS) was used to evaluate the risk level of perceptions, and the Drowning Risk Attitudes Scale (DRAS) was used to evaluate the risk level of attitudes.

**Results:**

The results of the mediating effect test showed that water safety knowledge (WSK) affected drowning risk behaviors (DRB) through three indirect paths. Drowning risk perceptions (DRP) and attitudes (DRA) have significantly mediated the association between WSK and DRB. In conclusion, DRP and DRA can act as mediators between WSK and DRB, not only individually, but also as chain mediators, where the direct effect is-0.301, the total indirect effect is-0.214, and the total mediated indirect effect is 41.5%.

**Discussion:**

Water safety knowledge negatively predicts adolescents’ drowning risk behaviors; water safety knowledge has an inhibitory effect on drowning risk perceptions. Water safety knowledge can directly influence adolescents’ drowning risk perceptions and indirectly affect drowning risk behaviors through the mediation of drowning risk perceptions and attitudes comprising three paths: (1) the drowning risk perceptions mediation path, (2) the drowning risk attitudes mediation path, and (3) the drowning risk perceptions and attitudes mediation paths.

## 1 Introduction

Drowning claimed more than 2.5 million lives worldwide from 2010 to 2019. According to the WHO’s most recent global health estimates, 236,000 people drowned in 2019, demonstrating that drowning deaths exceeded deaths from either protein-energy malnutrition or maternal conditions (1). Approximately 57, 000 people die from drowning annually in China, accounting for 20% of all drowning deaths worldwide. 56% of these deaths occur in children aged 5–14, in other words, 88 children die from drowning each day (2, 3). Therefore, it is crucial to understand how we can effectively prevent drowning among adolescents.

Drowning accidents of children and adolescents aged 5–14 are more likely to occur in open waters, such as rivers, lakes, ponds, reservoirs, and seas, owing to the hot summers resulting from global warming. Previous studies have found that the main causes of drowning accidents are drowning risk behaviors (DRB) and unintentional falls into water. Drowning risk behaviors refer to individuals putting themselves in risky circumstances on their own initiative, and unintentionally falling into open water refers to individuals’ risk ignorance (4). However, swimming skills and water safety knowledge (WSK) are key factors in these two causes. Mastering swimming skills and water safety knowledge is greatly significant to water safety education. Adolescents who acquire swimming skills may be able to overcome drowning, but without water safety knowledge, they still face the threat of drowning (3). Mastery of water safety knowledge not only changes adolescents’ drowning risk attitudes (DRA) and behaviors, but also strengthens their drowning risk perceptions (DRP). However, few studies have examined the mechanisms underlying water safety knowledge, drowning risk perceptions, drowning risk attitudes, and drowning risk behaviors.

Adolescents’ drowning risks are directly related to their drowning risk behaviors (5). A previous study found that individuals with a higher drowning risk tended to drown more than those with a low risk of drowning (6). Various types of water activities exist in China, including diving, snorkeling, water skiing, surfboard skiing, planking, catching fish, splashing, and swimming. Drowning accidents during these activities among adolescents are increasingly common because of the lack of guardian supervision and the induction of deviant behaviors from partners.

Mastering water safety knowledge helps adolescents reduce their drowning risk behaviors. Water safety knowledge consists of declarative knowledge about swimming safety and procedural knowledge about swimming rescue, including common sense of water safety, water safety legislative knowledge, and water safety judgments (5). (1) Common sense of water safety refers to a fundamental understanding of water activities, rescues, equipment, and the production of simple floating devices. (2) Water safety legislative knowledge refers to awareness of the laws and regulations of the water activity area, warning signs, beach flags, etc. (3) Water safety judgments refer to the ability to assess one’s own physical condition, weather, and water environment (4). In 2007, The International Lifesaving Federation stated that most drowning episodes can be prevented by understanding water safety and swimming skills (5). In general, a high level of water safety knowledge helps adolescents efficiently identify potential drowning risks. For example, adolescents would have better knowledge of various warning signs, could assess their own health, weather, and water conditions more effectively, and would use life-saving knowledge and skills to approach drowning events more reasonably. Moreover, water safety awareness and behaviors underpinned by water safety knowledge help avoid adolescents’ unintentional falls into open water, unintentional submersions, and drowning (4). Additionally, empirical studies have demonstrated that water safety knowledge strongly promotes pandemic prevention behaviors and health-related adaptive behaviors (7, 8), and prevents risky diving behaviors and unintentional injuries (9, 10). Based on this previous literature, we hypothesized that water safety knowledge may reduce the occurrence of drowning risk behaviors in adolescents.

*Hypothesis 1*: Water safety knowledge has an inhibition effect on drowning risk behavior.

**Figure 1:**
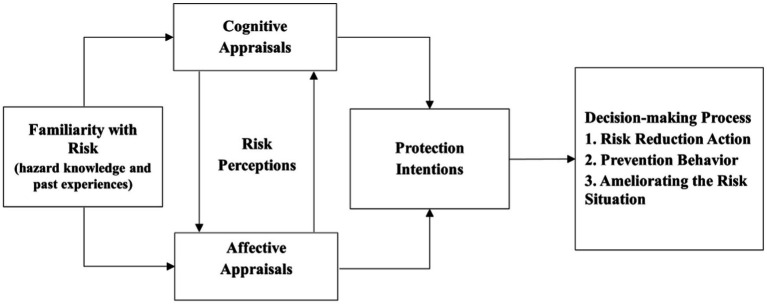
Conceptual illustration of the cognition-affect-intention (CAI) model.

In addition to directly influencing adolescents’ drowning risk behaviors, water safety knowledge can also indirectly prevent drownings by enhancing risk perceptions. Empirical research has demonstrated that the combination of water safety knowledge and risk perceptions has a substantial impact on drowning risk behaviors (8). Risk perceptions are used to describe people’s attitudes and intuitive judgments towards risk and play a crucial role in human safety behaviors (11). Altarawneha et al. (12) first applied a dual-process approach to the study of risk perception, arguing that public risk perception studies should combine cognitive and affective appraisals to form a dynamic “dual-process” (Figure 1). Risk perceptions are significantly related to the level of subjective knowledge (risk awareness) and the degree of personal familiarity with water environments; thus, they can drive individuals’ behavioral decisions (12). Furthermore, the dual-process approach model also supports Finucane’s point that “understanding how affect and cognition interact and collaborate in human judgment and decision-making is crucial for understanding risk perceptions” (13). Adolescents’ understanding of water environments’ risks originate from their water safety knowledge. For example, recognizing water safety signs and beach safety flags may warn adolescents to inhibit some drowning risk behaviors (4). Moreover, water safety knowledge enhances adolescents’ drowning risk perceptions, alerts them to drowning risks, and encourages persuasion and precautions against drowning risk behaviors. However, it is unclear whether water safety knowledge inhibits drowning risk behaviors through drowning risk perceptions. Accordingly, we predicted that drowning risk perceptions would have an inhibition role in the relationship between water safety knowledge and drowning risk behaviors (14, 15).

*Hypothesis 2*: Drowning risk perceptions have an inhibition role in the relationship between water safety knowledge and drowning risk behaviors.

The Knowledge, Attitude, and Practice (KAP) theory commonly utilized to study people’s health behavior divides human behavior change into three sequential processes: (1) acquiring knowledge, (2) forming beliefs and positive attitudes, and (3) eventually changing behaviors. In this process, knowledge is the basis for behavioral change, while beliefs and attitudes are the forces behind behavior change (16). Moreover, the KAP model is one of the most effective methods for drowning prevention because it considers people’s existing knowledge, beliefs, local environments, and social norms to understand why they engage in risky drowning behaviors (4). However, a study of young surfers in New Zealand found that surfing safety knowledge and attitudes had a significant impact on their high-risk surfing behaviors, which contributed to their high drowning probability (17). Many studies have demonstrated that the application of KAP theory to mediation models is a reliable predictor of drowning risk for all types of water activities, and that attitudes are important as a mediating variable between knowledge and behavior (15, 18, 19). Therefore, we posit that drowning risk attitudes can work as a mediator in the relationship between water safety knowledge and drowning risk behaviors.

*Hypothesis 3*: Drowning risk attitudes will mediate the relationship between water safety knowledge and drowning risk behaviors.

**Figure 2:**
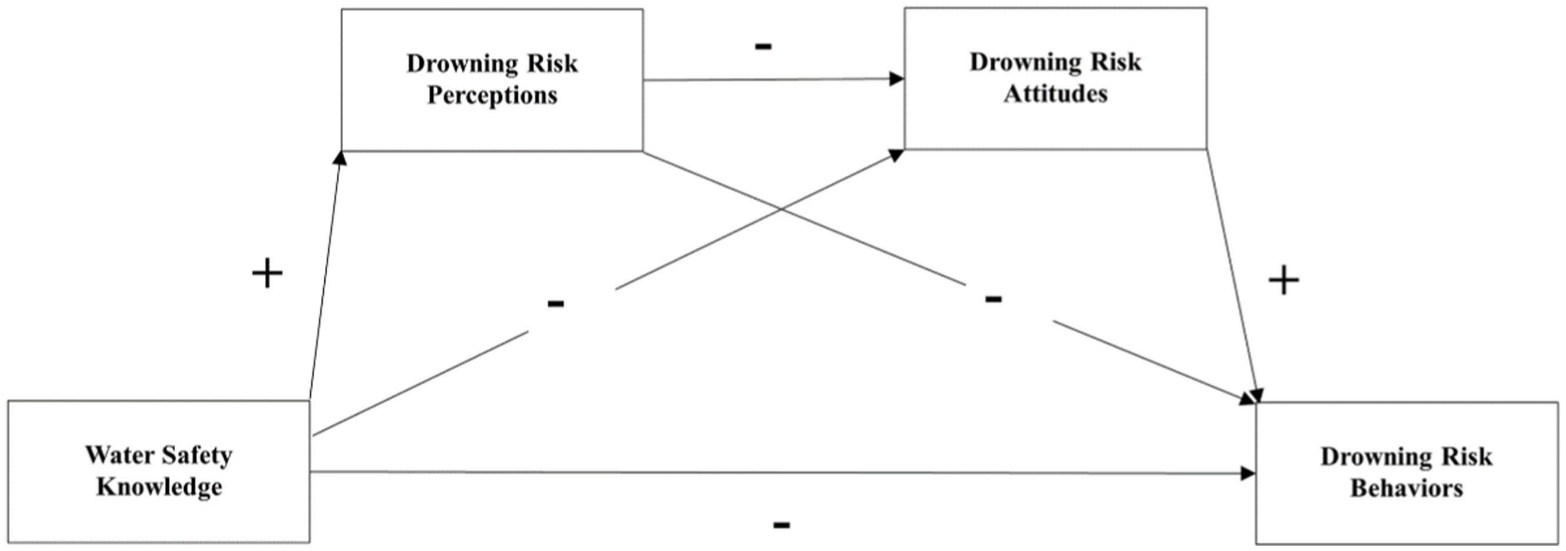
Theoretical model.

The Knowledge, Risk perception, Attitude, and Practice (KRPAP) theoretical model was created by adding risk perception factors based on the KAP theory. It is conceivable to investigate the potential chain mediation of risk perceptions and attitudes on drowning risk behavior using the KRPAP theory, which holds that people’s behavior undergoes a knowledge-risk perceptions-attitudes-behavior change process. Taking the COVID-19 pandemic as an example, factors such as the administration’s inability to release pertinent information adequately and in a timely manner, the public not being well informed about COVID-19, and information asymmetry all might result in erroneous risk perceptions. This impact has the potential to transform people’s attitudes and behaviors over time, leading to verbal and behavioral radicalization among the public and contributing to societal instability (20). Compared with the KAP model, the KRPAP model has been used less frequently in previous drowning prevention research. Based on the above analysis, we hypothesized that drowning risk perceptions and attitudes would have a chain mediating effect between water safety knowledge and drowning risk behaviors (Figure 2).

*Hypothesis 4*: Drowning risk perceptions and attitudes have a chain-mediating effect on water safety knowledge and drowning risk behaviors.

## 2 Materials and methods

### 2.1 Participants and procedure

We used cross-sectional data from primary and secondary school students in China, and conducted pre-test training for local teachers and students to normalize the investigation process. Before completing the formal questionnaire, all primary and secondary school students agreed to participate, and written informed consent forms were obtained from their primary guardians. We distributed and obtained 8,000 questionnaires, of which 7,485 (93.6%) were valid. Finally, this study included 3,663 males (48.9%) and 3,822 females (51.1%). Ethical approval was obtained from the Institutional Review Board of the College of Physical Education at Southwest University (ethical approval number: SWU20180601).

### 2.2 Measures

#### 2.2.1 Drowning risk behaviors scale

The Drowning Risk Behaviors Scale (DRBS) was used to evaluate an individual’s risk level for drowning behavior (15). The Chinese version of the DRBS is considered to have good reproducibility and validity (21). The drowning risk behaviors scale consisted of 10 items (e.g., “Swimming with no adults”; “Swimming in the wild waters without safety protection”; “Diving in the unknown depth of water”; “Playing roughshod with your mates while swimming”; “Swimming when you are sick”) rated on a five-point Likert scale ranging from 1 (Never) to 5 (Always). All items were added to produce a total score, with higher scores indicating a more severe drowning risk. The internal consistency coefficient (Cronbach’s alpha) was 0.934.

#### 2.2.2 Drowning risk perceptions scale

The Drowning Risk Perceptions Scale (DRPS) was used to evaluate an individual’s level of drowning risk perceptions (22). The reliability and validity of the Chinese version of the DRPS have been previously described (6). DRPS consists of 13 items and four subscales, including F1 = Susceptibility to drowning (e.g., “Drowning is a leading cause of death among children”; “Every student is at the risk of drowning”; “You and your partners are at the risk of drowning”), F2 = Seriousness of drowning (e.g., “Drowning is a serious problem”; “Drowning students must be taken to hospital”; “Most drowning victims would die”). F3 = Benefits of swimming skills (e.g., “Swimming is a life skill”; “Swimming skills will decrease drowning risk”; “Swimming skills are necessary for a particular professional career”), and F4 = Barriers perceived (e.g., “Lack of swimming instructors”; “Lack of swimming lessons in school”; “Unaffordable swimming lessons”; “Swimming pool is too far”). Each item was rated on a five-point Likert scale ranging from 1 (strongly disagree) to 5 (strongly agree). All items were summed to produce a total score, with higher scores indicating a higher drowning risk perception. In this study, the internal consistency reliability (Cronbach’s alpha) of the overall scale was 0.839, and the internal consistency coefficients (Cronbach’s alpha) of the four-dimensional scales were 0.703, 0.788, 0.746, and 0.720. From the degree of fit of the four-factor model, CFI, IFI, NNFI, and GFI >0.90, and RMSEA <0.08, indicating that the four-factor structural model fits well.

#### 2.2.3 Water safety knowledge scale

We used the Water Safety Knowledge Scale (WSKS) to evaluate an individual’s level of water safety knowledge (23). The Chinese version of the WSKS has good reproducibility and validity (6). The water safety knowledge scale consisted of 10 items: (1) “Do you know about water safety?”; (2) “Do you know the common methods for rescuing those who fall into water?”; (3) “Do you know how to perform self-rescue in water?”; (4) “Do you know how to perform CPR?”; (5) “Can you recognize common water safety signs?”; (6) “Do you know how to react when someone else falls into the water?”; (7) “Do you know the correct use of life jackets and life preservers?”; (8) “Do you know the most effective ways to call for help while drowning?”; (9) “Do you know how to rest when fatigued by swimming?”; and (10) “Do you know how to swim safely?.” These were rated on a five-point Likert-type scale ranging from 1 (very unfamiliar) to 5 (very familiar). All items were summed to produce a total score, with higher scores indicating a higher level of education. The internal consistency coefficient (Cronbach’s alpha) was 0.943.

#### 2.2.4 Drowning risk attitudes scale

The Drowning Risk Attitudes Scale (DRAS) was used to evaluate an individual’s drowning risk attitudes. The Chinese version of the DRA has good reproducibility and validity (23). The DRAS consists of 10 items: (1) “Before swimming, is there no need to consider whether the waters are safe?”; (2) “Are good swimmers always safe from drowning?”; (3) “Is swimming in rivers safe?”; (4) “Is it best to enter water immediately to help your partner draw?”; (5) “Does swimming in shallow water guarantee that you will not drown?”; (6) “Is it safe to swim with a partner who can swim, instead of an adult?”; (7) “Is it always safe to go swimming with a lifejacket?”; (8) “Is it safe to play near the edge of water without entering it?”; (9) “Is walking on ice safe?”; and (10) “Is it safe to swim while wearing clothing?”. These were rated on a five-point Likert-type scale ranging from 1 (strongly disagree) to 5 (strongly agree). All items were summed to produce a total score, with higher scores indicating higher levels of risk intention. The internal consistency coefficient (Cronbach’s alpha) was 0.964.

### 2.3 Data analysis

SPSS version 26.0 was used to explore the correlations among water safety knowledge, drowning risk perceptions, drowning risk attitudes, and drowning risk behaviors. Harman’s single-factor test was utilized to examine 42 items for the common method bias test (24). The results revealed 11 factors with eigenvalues greater than one, and the variance explained by the first factor was less than 40% (19.246%), indicating that there was no common method bias in this study.

Mediating hypotheses were examined based on the mediating effects analysis process (25). All data were analyzed using Hayes’ SPSS macro program PROCESS (26). Water safety knowledge was used as an independent variable, drowning risk perceptions and attitudes as mediating variables, and drowning risk behaviors as dependent variables. The mediating effect was tested using bootstrapping (repeated sampling of 5,000 times). If the 95% confidence interval did not include zero, the mediating effect was considered significant.

## 3 Results

### 3.1 Descriptive and Pearson correlation analysis

The results of the correlation analyses for all variables are shown in Table 1. Drowning risk perceptions are positively correlated with water safety knowledge; drowning risk attitudes and behaviors are negatively correlated with water safety knowledge; drowning risk attitudes and behaviors are negatively correlated with drowning risk perceptions; and drowning risk behaviors are positively correlated with drowning risk attitudes.

**Table 1:** Descriptive statistics and correlations of key variables (*n* = 7,485).

	M ± SD	1	2	3	4
1. Water safety knowledge	2.35 ± 0.86	—			
2. Drowning Risk Perceptions	3.07 ± 0.88	0.371^**^	**—**		
3. Drowning Risk Attitudes	2.49 ± 0.87	−0.307^**^	−0.377^**^	**—**	
4. Drowning Risk Behaviors	2.48 ± 0.85	−0.445^**^	−0.259^**^	0.485^**^	**—**

### 3.2 Testing for chain mediating effect

The results of the regression analysis are presented in Table 2. Water safety knowledge positively predicted drowning risk perceptions (β =0.371, *p*< 0.001). Water safety knowledge and drowning risk perceptions negatively predicted drowning risk attitudes (β = −0.194, *p* < 0.001; β = −0.305, *p* < 0. 001). When incorporating water safety knowledge, drowning risk perceptions and drowning risk attitudes into the equation, their predictive effects were significant. Namely, water safety knowledge and drowning risk perceptions negatively predicted drowning risk behaviors (β = −0.260, *p* < 0. 001; β = −0.213, *p* < 0. 001), and drowning risk attitudes positively predicted drowning risk behaviors (β =0.345, *p* < 0.001).

**Table 2:** Regression analysis of the mediation model between drowning risk perceptions and drowning risk attitude.

Independent variable	Model 1 (Dependent variable:DRP)			Model 2 (Dependent variable:DRA)			Model 3 (Dependent variable:DRB)		
	β	SE	*t*	β	SE	*t*	β	SE	*t*
WSK	0.371	0.012	31.657	−0.194	0.011	−17.078	−0.260	0.010	−24.873
DRP				−0.305	0.012	−25.227	−0.213	0.012	−18.285
DRA							0.345	0.011	31.608
R^2^		0.02			0.03			0.15	
F		15.839			17.946			33.528	

**Figure 3:**
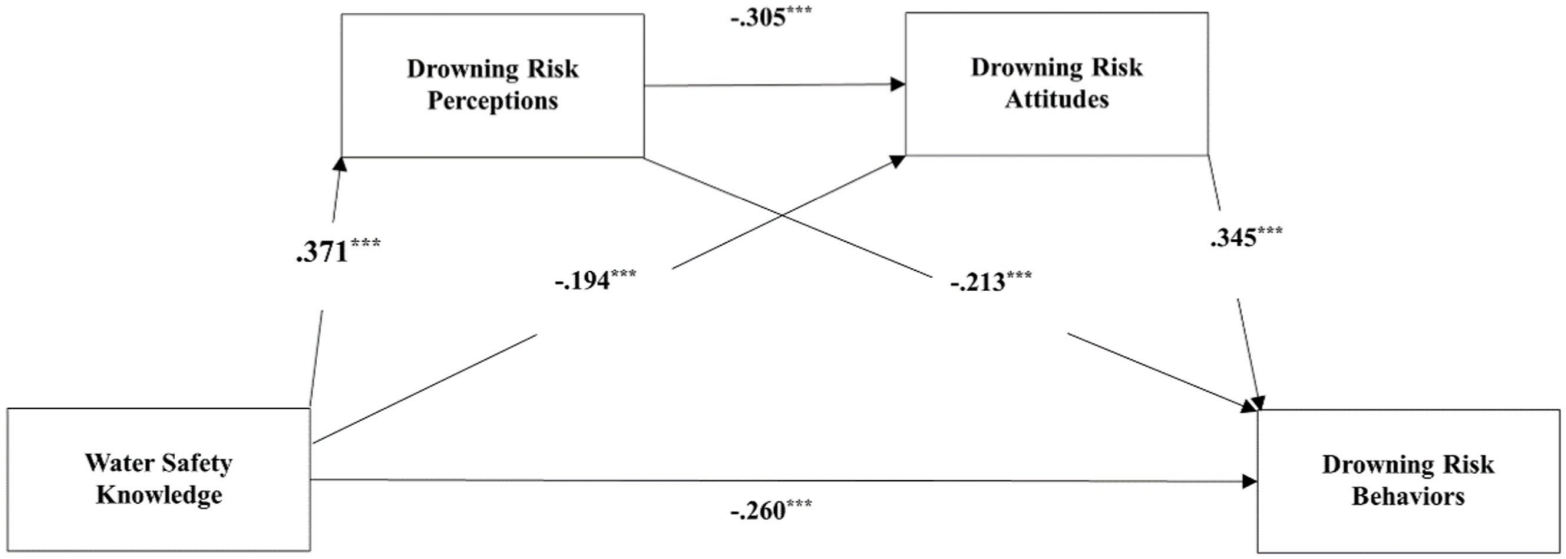
The chain mediating model. Path coefficients are standardized coefficients. ^***^p < 0.001.

The results of the mediating effects tests are presented in Table 3 and Figure 3. WSK affected DRB through three indirect paths. The 95% confidence interval (CI) of the indirect effect paths did not include zero, revealing that DRP and DRA had significant mediating effects on the association between WSK and DRB. The first path, WSK → DRP → DRB, accounts for 17.7% of total effect. The second path, WSK → DRA → DRB, accounted for 15.1% of the total effect. The third path, WSK → DRP → DRA → DRB, accounted for 8.7% of the total effect. In conclusion, DRP and DRA can act as mediators between WSK and DRB, not only individually, but also as chain mediators, where the direct effect is-0.301, the total indirect effect is-0.214 (a1b1 + a2b2 + a1a3b2), and the total mediated indirect effect is 41.5%.

**Table 3:** Mediation effect volume analysis.

	Indirect effect value	Boot SE	95%CI	Effect amount
Direct Effect	−0.301	0.013	[−0.328, −0.274]	58.5%
WSK → DRP → DRB(a1*b1)	−0.091	0.006	[−0.104, −0.079]	17.7%
WSK → DRA → DRB(a2*a2)	−0.078	0.006	[−0.089, −0.067]	15.1%
WSK → DRP → DRA → DRB(a1*a3*b2)	−0.045	0.003	[−0.052, −0.039]	8.7%
Total Mediation Effect	−0.214	0.011	[−0.235, −0.193]	41.5%
Total Effect	−0.515	0.014	[−0.543, −0.487]	

## 4 Discussion

The resolution adopted by the General Assembly on April 28, 2021 stresses the need to raise awareness of the importance of drowning prevention and the need for urgent coordinated multisectoral action to improve water safety through education, knowledge sharing, and other activities, with the aim of reducing preventable deaths (27). Our results support the role of water safety knowledge as a significant negative predictor of drowning risk behaviors and are consistent with previous findings (28, 29). Adolescents who have a high level of water safety knowledge can be sensitive to physical conditions, weather, and potential risks of aquatic environments, and can combine the skills they have learned to save themselves and others. For instance, adolescents’ familiarity with water warning signs will enable them to reduce their drowning risk.

China currently ranks first in the world in terms of drowning fatalities, and water safety education lags severely. We found that few primary and secondary schools had teaching materials related to the recognition of water warning signs. It is noteworthy that the proportion of schools and universities with swimming pools was relatively low. Taking the nine top economic districts in Chongqing as an example, only 8 of the 446 (1.79%) primary schools, 18 of the 249 (7.23%) middle schools, and 12 of the 71 (16.9%) universities were equipped with swimming pools. In this context, two factors are extremely important for promoting the rapid development of water safety education in China. The first factor is parental involvement. Morrongiello et al. (30) found that, in addition to schools, parents were an especially critical element in the development of adolescents’ drowning prevention skills, which was even more apparent in China. Some parental approaches, such as enrolling children in aquatics training, are the most beneficial in helping children develop the water safety knowledge and swimming skills (31). The second most important factor is the financial support. The WHO (3) has noted that more than 90% of drowning fatalities occur in low-and middle-income countries (LMIC). However, certain urban and rural parts of China can be compared with both developed countries and LMICs. There are families in China’s rural areas whose parents earn little income and cannot cover the costs for their children to receive water safety education. Therefore, financial support can help reduce the incidence of drowning accidents by providing more adolescents with opportunities to receive water safety education.

Our results support the inhibitory effect of water safety knowledge on drowning risk behaviors through drowning risk perceptions. Meanwhile, regarding Hypothesis 2 of this study, the second path (WSK → DRA → DRB) is consistent with previous research findings (32) while the first path (WSK → DRP → DRB) is our new finding. However, water safety knowledge strengthens adolescents’ drowning risk perceptions and can thus decrease the probability of adolescents intentionally exposing themselves to risk. Moreover, the dual-process approach model indicated that risk perceptions were significantly related to subjective knowledge and personal familiarity with the water environment, which in turn, helped adolescents avoid drowning risk behaviors. More specifically, individuals develop the ability to identify risk through the collection, comprehension, and application of knowledge, and build up their “antibodies” to self-protect against drowning. These “antibodies” are a stress response to water environments and enable one to remain alert to threats in the aquatic environment.

However, sensitivity to the aquatic environment and the ability to be aware of potential drowning risks in advance also rely on mastering water safety knowledge. Furthermore, a combination of knowledge and risk perception is also essential, especially when drowning prevention and rescue of oneself or others requires the most appropriate judgement and decision-making. However, unintentionally falling into the water and drowning requires extra attention (33). In China, most children and adolescents do not know how to swim or are not good swimmers. According to a vast number of drowning accidents reported, diving, swimming in deep water, and physical exhaustion are the leading causes of drowning in adults, whereas unintentional falls into water are more common among children and adolescents (2). In the case of unintentional drowning, it is difficult for a drowning adolescent or adult to survive because of heavy and wet clothing reducing the speed of swimming, countercurrents, and other factors (34). The strengthening of drowning precautions and the improvement of risk perception and risk identification stem from the continuous learning of water safety knowledge, so that the drowning risk can be reduced.

In this study, we found that drowning risk attitudes partially mediated the relationship between water safety knowledge and drowning risk behaviors, which is consistent with previous studies (15, 17). The use of KAP theory in drowning prevention research has been well established, and the WHO (4) has also specifically highlighted its use of KAP theory (4). In China, the Ministry of Education, schools, and students are the three key participants in the implementation of water safety education. First, the Ministry of Education plays a general directive role in drowning prevention education in schools by establishing a dedicated educational website and propagating drowning prevention on its official website. Schools are more direct implementers of water safety education, primarily through awareness campaigns and lectures just before summer holidays, as well as through public education on WeChat. However, owing to academic pressure and the paucity of teachers and teaching equipment, water safety courses have limited class time and are not as popular as they should be. Finally, students, who are the target audience for water safety education, participate in courses and lectures to gain water safety knowledge. Nevertheless, many students lack motivation to attend water safety education courses and learn very little about water safety knowledge simply because these courses are not part of the main academic assessment system for students.

In summary, the water safety education program in Chinese schools lacks organization, resulting in a low level of adolescent water safety knowledge and passive drowning risk attitudes. Negative attitudes invariably lead to negative behaviors, which can exacerbate issues such as drowning accidents, shortsightedness, and deteriorating health. Negative attitudes towards health-harming behaviors generated by exam-oriented education tend to result in negative behaviors and exacerbate drowning accidents, shortsightedness, deteriorating physical fitness, and other issues that the Chinese society must address.

Our study also demonstrated that drowning risk perceptions and attitudes act as chain mediators between water safety knowledge and drowning risk behaviors. An increase in water safety knowledge promotes drowning risk perceptions, which in turn can improve drowning risk attitudes and prevent risky behaviors. This pathway, which has not been extensively explored in previous drowning prevention studies, is consistent with findings on the prevention of heat-related illnesses (35). Negative information refers to information that threatens individuals’ safety and sensitizes them to environmental dangers. However, owing to the limited knowledge volume, water safety knowledge does not contain much negative information. This may easily lead adolescents to lose awareness of water environment dangers, resulting in negative attitudes and riskier behaviors. Chinese transportation authorities have attempted to include negative information in dangerous driving education, such as the use of vehicle accident videos, to make drivers more alert of risky behaviors including drink driving, speeding, and running red lights.

In drowning prevention studies, a similar strategy can be employed to raise awareness of the serious consequences of drowning accidents to prevent high-risk rescue behaviors, such as direct diving, hand-holding, and direct hand rescuing by adolescents. Summarizing and learning from other people’s drowning experiences can help individuals avoid mistakes. Moreover, according to psychological education, we should ensure that the warning bells are always ringing, because risk perceptions, like knowledge and memory, will diminish with time and the influence of other people and events. Currently, China’s birth population is decreasing each year, and the deaths of adolescents due to drowning accidents pose a threat to the young population. Therefore, more emphasis should be placed on water safety education to protect young people as well as the future of families and the country at large.

## 5 Limitations and future research

This study has several limitations. First, this study followed a cross-sectional design; therefore, we could not establish the causal association between water safety knowledge and drowning risk behaviors. Cross-lagged designs and experimental interventions are needed in future studies. Second, this study was limited to exploring the mediating effect of drowning risk perceptions and attitudes on adolescents’ water safety knowledge and drowning risk behaviors; whether other factors, such as sensation seeking, emotion, swimming overconfidence, verbal persuasion, and behavioral imitation, also play a moderating role needs further examination. Third, future studies may need to apply water competency models and Behavioral Event Interviews (BEI) to identify the characteristics of various water competencies and behaviors to better prevent adolescent drowning accidents.

## 6 Conclusion

Water safety knowledge negatively predicts adolescent drowning risk behaviors, and has an inhibitory effect on drowning risk perceptions. Water safety knowledge can directly influence adolescents’ drowning risk perceptions and indirectly affect drowning risk behaviors through the mediation of drowning risk perceptions and attitudes comprising three paths. The first was the drowning risk perceptions mediation path. Second was a drowning risk attitudes mediating path. The third chain mediation path involved drowning risk perceptions and attitudes. In conclusion, the establishment of the mediation model reveals the mechanism by which water safety knowledge influences adolescents’ drowning risk behaviors, serving as a reference for the prevention of adolescent drowning risk behaviors. In the future, the level of water safety knowledge can be improved to help adolescents enhance their drowning risk perceptions, avoid negative risk attitudes, and reduce drowning risk behaviors.

## Data availability statement

The raw data supporting the conclusions of this article will be made available by the authors, without undue reservation.

## Ethics statement

The studies involving humans were approved by the Institutional Review Board of the College of Physical Education at Southwest University (ethical approval number: SWU20180601). The studies were conducted in accordance with the local legislation and institutional requirements. Written informed consent for participation in this study was provided by the participants' legal guardians/next of kin. Written informed consent was obtained from the minor(s)' legal guardian/next of kin for the publication of any potentially identifiable images or data included in this article.

## Author contributions

ShiL: Writing – review & editing, Writing – original draft, Visualization, Validation, Supervision, Software, Resources, Project administration, Methodology, Investigation, Funding acquisition, Formal analysis, Data curation, Conceptualization. ShuL: Writing – review & editing. ZR: Writing – review & editing, Writing – original draft, Visualization, Validation, Supervision, Software, Resources, Project administration, Methodology, Investigation, Formal analysis, Data curation, Conceptualization. HZ: Funding acquisition, Writing – review & editing, Validation, Supervision. XL: Writing – review & editing. LL: Writing – review & editing.
